# The role of platelet desialylation as a biomarker in primary immune thrombocytopenia: mechanisms and therapeutic perspectives

**DOI:** 10.3389/fimmu.2024.1409461

**Published:** 2024-06-24

**Authors:** Qianhui Zhang, Miao Huang, Elizabeth Rosalind Thomas, Lu Wang, Jia Liu, Xiang Li, Jiesi Luo, Wenjun Zou, Jianming Wu

**Affiliations:** ^1^ State Key Laboratory of Southwestern Chinese Medicine Resources, Chengdu University of Traditional Chinese Medicine, Chengdu, China; ^2^ School of Basic Medical Sciences, Southwest Medical University, Luzhou, China; ^3^ Department of Microbiology, North Eastern Indira Gandhi Regional Institute of Health and Medical Sciences, Shillong, India; ^4^ Education Ministry Key Laboratory of Medical Electrophysiology, Medical Key Laboratory for Drug Discovery and Druggability Evaluation of Sichuan Province, Luzhou Key Laboratory of Activity Screening and Druggability Evaluation for Chinese Materia Medica, Luzhou, China

**Keywords:** platelets, sialic acid, primary immune thrombocytopenia, pathogenesis, apoptosis

## Abstract

Primary immune thrombocytopenia (ITP) is an acquired autoimmune disorder characterized by the destruction of platelets. Although it was long believed that the critical role of autoantibodies in platelet destruction, primarily through the Fc-dependent platelet clearance pathway, recent findings indicate that the significance of the Fc-independent platelet clearance pathway mediated by hepatocytes, thus shedding light on a previously obscure aspect of ITP pathogenesis. Within this context, the desialylation of platelets has emerged as a pivotal biochemical marker. Consequently, targeting platelet desialylation emerges as a novel therapeutic strategy in the pathogenesis of ITP. Notably, prevailing research has largely focused on antiplatelet antibodies and the glycosylation-associated mechanisms of platelet clearance, while comprehensive analysis of platelet desialylation remains scant. In response, we retrospectively discuss the historical progression, inducing factors, generation process, and molecular regulatory mechanisms underlying platelet desialylation in ITP pathogenesis. By systematically evaluating the most recent research findings, we contribute to a comprehensive understanding of the intricate processes involved. Moreover, our manuscript delves into the potential application of desialylation regulatory strategies in ITP therapy, heralding novel therapeutic avenues. In conclusion, this manuscript not only fills a critical void in existing literature but also paves the way for future research by establishing a systematic theoretical framework. By inspiring new research ideas and offering insights into the development of new therapeutic strategies and targeted drugs, our study is poised to significantly advance the clinical management of ITP.

## Graphical Abstract

The role of platelet desialylation in primary immune thrombocytopenia.

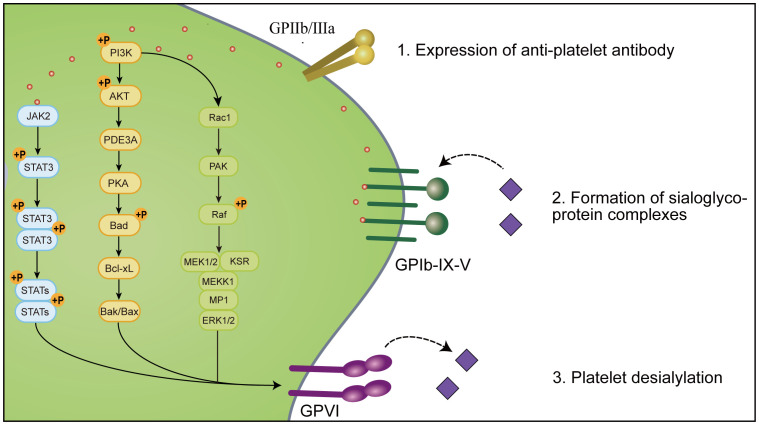

## 1 Introduction

Primary immune thrombocytopenia (ITP), an acquired autoimmune bleeding disorder, displays diverse characteristics of heterogeneity (1), chronicity (2), asymptomatic nature (3), and severity (4). As the global population ages, there is a discernible increase in the quantity of ITP cases. The reported incidence of primary ITP ranges from 1.9 to 6.4 per 100,000 per year in children (5) and 2 to 10 per 100,000 per year in adults (6). Notably, there is a higher prevalence among individuals over 60 years of age, contributing to the growing health concern associated with this condition. Additionally, women of childbearing age exhibit a slightly elevated prevalence compared to men in the same age group, with rates ranging approximately from 9.5 to 23.6 per 100,000 per year (7). Beyond the physical manifestation, patients grappling with ITP face not only the imminent risk of severe or fatal bleeding events but also content with a myriad of challenges, including a high disease burden and unfavorable prognosis. These factors contribute significantly to mental stress and psychological burden. A retrospective analysis highlights the economic impact, revealing that drug therapy for ITP incurs costs in hundreds of millions of dollars annually, translating to an average yearly expense of approximately $28,000 per ITP patient in the United States (8). Despite notable advancements in ITP treatment, the disease’s incidences remain elevated, and there exists a lack of standardization in both diagnosis and treatment protocols. Currently, there are no standardized guidelines for the diagnosis and treatment of ITP, which is a diagnosis of exclusion. This means that the disease is identified by the absence of other causes or conditions that could explain the reduction in platelet count. In addition to reduced platelets, the rest of the blood cell counts are normal. The diagnosis of ITP can only be confirmed by other secondary factors of thrombocytopenia except medical history, physical examination, blood count, and peripheral blood smear microscopy (9–11). Nevertheless, this limited approach introduces a risk of misdiagnosis, underscoring the critical need for precise clinical diagnosis and individualized treatment strategies. Addressing these challenges is imperative for enhancing patient outcomes and refining the overall management of ITP.

However, a lack of a clear understanding of the pathogenesis of ITP directly hinders the attainment of precise diagnosis and personalized treatment strategies. Current research suggests that the pathogenesis may be linked to immune system dysregulation and the generation of autoantibodies targeting platelets. In other words, the body’s immune system erroneously identifies its platelets as foreign substances, leading to the production of antibodies that attack these platelets. This process results in excessive platelet destruction and clearance (12). Prior investigations have proposed that platelet destruction caused by platelet autoantibodies primarily occurs through the Fc-dependent platelet clearance pathway (FcγR). In this pathway, the Fc segment of autoantibodies in ITP patients binds to the Fc receptor on the surfaces of macrophages or hepatic macrophages (Kupffer cells) located in the hepatosplenic reticuloendothelial system. This forms an antibody-receptor complex triggering platelet destruction and clearance by immune cells. Simultaneously, it inhibits platelet aggregation and adhesion, ultimately leading to thrombocytopenia (13, 14). Apart from the platelet destruction mediated by FcγR, recent research has shown a growing interest in hepatocyte-mediated Fc-independent platelet clearance pathway. In this process, platelet autoantibodies induce the desialylation of sialic acid from the platelet surface. Desialylated platelets are then recognized and cleared by the Ashwell-Morell receptor (AMR) present in hepatocytes (15, 16). Platelet desialylation emerges as a critical biochemical marker influencing both platelet count and functionality. Mounting evidence indicates that platelet desialylation plays a pivotal role in ITP pathogenesis, with the process of platelet destruction likely correlated with the production of platelet desialylation and its molecular regulatory mechanisms (17–19). Subsequent observations revealed a gradual recovery in peripheral platelet counts in mice as sialidase levels increased (16). Clinical studies demonstrated that patients with infection-induced thrombocytopenia exhibit significantly higher levels of platelet desialylation (16, 20). Additionally, the use of the sialidase inhibitor Oseltamivir to inhibit platelet desialylation could significantly alleviate the phenomenon of low platelet counts (21, 22), thus suggesting a broader mechanism of platelet desialylation. Altered desialylation in patients with ITP not only impairs platelet count and functionality but also induces increased apoptosis, as evidenced by heightened caspase-9 activation, particle formation, and a reduction in platelet lifespan. These factors collectively contribute to a decrease in platelet count and compromised function (23–26). The aforementioned discoveries suggest that the liver’s phagocytosis of desialylated platelets signifies a major pathway for platelet breakdown, significantly contributing to the development of ITP.

Therefore, in this manuscript, we synthesized the latest domestic and international research findings to delineate the historical progression of platelet desialylation and its triggering factors. We subsequently conducted an in-depth analysis of the generation process of platelet desialylation and its molecular regulatory mechanisms. Finally, we explore the potential applications of sialylation regulation strategies in the treatment of ITP. Furthermore, we propose some prospective views and strategic recommendations that can inform the clinical treatment of ITP by elucidating the role of platelet desialylation in the pathogenesis of the condition. These recommendations include methods to reduce viral and bacterial infections, along with their associated antigenic stimulation of the immune system. We also explore approaches to modulate the expression of anti-platelet antibodies and the formation process of sialic acid-glycoprotein complexes. Additionally, we delve into the investigation of specific targets and biomarkers associated with platelet desialylation to mitigate its impact on platelet function. The overarching goal of these efforts is to offer more beneficial targets and strategies for clinical management and drug development of ITP, to avoid unnecessary or ineffective treatments. By facilitating a precise diagnosis and individualized treatment for ITP patients, we aspire to enhance treatment outcomes and improve the quality of life for those affected by ITP.

## 2 Historical progression of platelet desialylation

**Figure 1:**
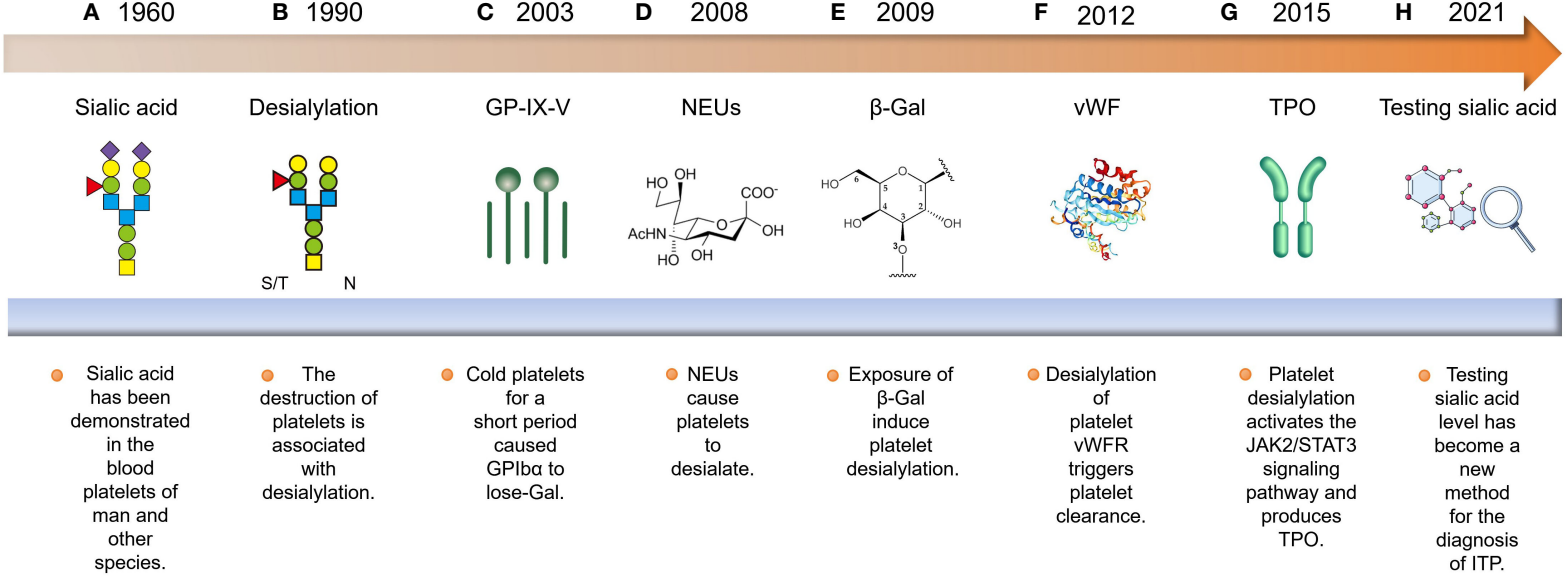
Historical progression of platelet desialylation: **(A, B)** The discovery of platelet desialylation can be traced back to the 1960s. **(C)** Subsequently, research has elucidated its significant impact on the pathogenesis of ITP, particularly in relation to the exposure of GPIbα to N-GlcNAC recognition sites, which are cleared by αMβ_2_ recognition. **(D, E)** Furthermore, researchers found that neuraminidases released by *Streptococcus pneumoniae* caused platelet desialylation, exposing β-Gal. This exposed residue is recognized and cleared by the AMR. **(F)** Additionally, desialylation of the vWFR on platelets accelerated their clearance. **(G, H)** With an in-depth understanding of the mechanisms governing platelet desialylation, relevant diagnostic and therapeutic methods have emerged. These include sialic acid level detection, immunosuppressant administration, and immunoglobulin therapy.

Over the past few decades, significant breakthroughs have been made in understanding the process of platelet desialylation and its impact on the development and progression of hematological diseases (**Figure 1**). Research from as early as the 1960s first noted that platelets in ITP patients had reduced sialic acid content, leading to their excessive destruction and clearance (17). This observation sparked in-depth studies into the mechanisms of platelet desialylation, particularly the Fc-independent pathways of platelet clearance. In 2003, Hoffmeister et al. made a groundbreaking discovery, revealing that platelets stored *in vitro* and then transfused into humans were quickly cleared by the liver. Further studies revealed that platelets stored for less than 2 hours lost β-galactose (β-Gal) on the membrane glycoprotein GPIbα, thereby exposing N-acetyl glucosamine (N-GlcNAc) recognition sites. This led to the rapid recognition and clearance of these platelets by αMβ_2_ macrophages in the liver (27, 28). This finding answered long-standing questions about why stored platelets were rapidly cleared after transfusion. However, subsequent studies showed that blocking the N-GlcNAc/αMβ_2_ pathway did not resolve the issue of liver clearance of stored platelets. A breakthrough came in 2008 when Grewal found that pneumococcal-induced sepsis led to the release of neuraminidase (NEUs) by the bacteria, which caused platelet desialylation and exposed β-Gal, allowing the platelets to be recognized and cleared by the AMR (29). This discovery not only shed light on a novel function of the AMR but also offered a fresh perspective on the platelet clearance pathway’s mechanism. Studies also indicated that long-term storage (over 48 hours) of platelets led to the recognition of β-Gal on GPIbα by the AMR, potentially leading to platelet desialylation (18, 30, 31). Additionally, desialylation of the von Willebrand factor (vWF) receptor on platelets accelerated their clearance (32). In 2015, Li et al. found that anti-GPIbα antibodies in ITP patients trigger the surface translocation of NEU1, leading to platelet desialylation and subsequent recognition and clearance by the AMR. This process could be blocked by sialidase inhibitors (15). Further research discovered that the binding of desialylated platelets to the AMR was discovered to activate the JAK2/STAT3 signaling pathway, promoting the production of thrombopoietin (TPO) production and thereby regulating thrombopoiesis (33). With an improved understanding of platelet desialylation mechanisms, researchers have developed more accurate diagnostic methods and therapeutic approaches, including sialic acid level testing (34), immunomodulators (35), immunoglobulin therapies (36), and a few novel medications (37). These advancements aim to alleviate symptoms and enhance the quality of life for patients with ITP.

Hence, reviewing the research on platelet desialylation not only provides references for the treatment of ITP but also offers hope for enhancing the quality of life for these patients.

## 3 Inducing factors for platelet desialylation

**Figure 2:**
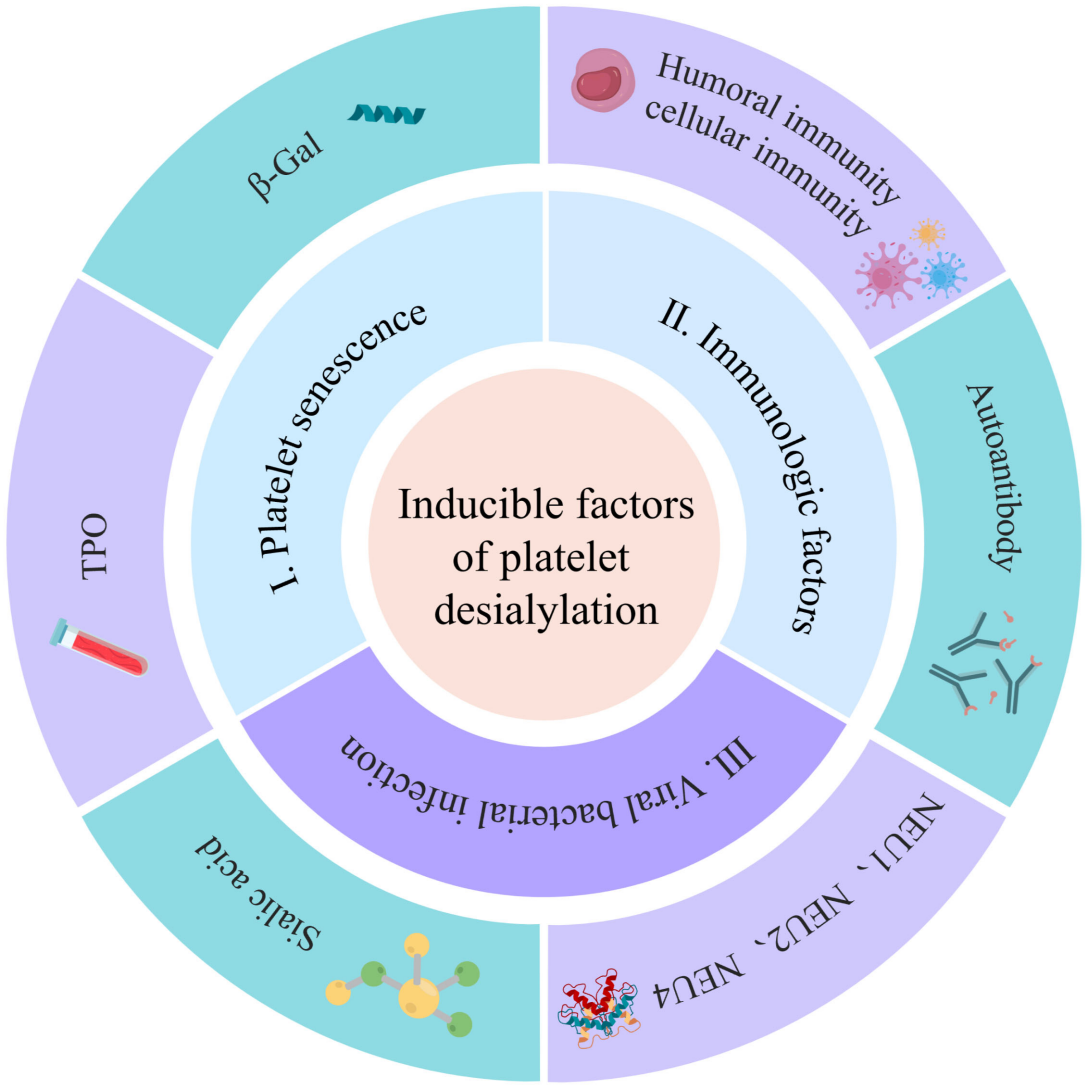
Inducing factors for platelet desialylation: The factors contributing to platelet desialylation include immune factors, viral and bacterial infection, and platelet senescence. The immune factors predominantly encompass platelet autoantibodies, humoral immunity, and cellular immunity. Additionally, foreign antigens and self-platelet antigens carried by the viruses can trigger cross-activation of B cells and T cells, leading to epitope spreading and subsequent platelet desialylation. Simultaneously, pathogen-derived NEUs serve as pivotal regulators, contributing significantly to the induction of platelet desialylation.

The factors contributing to platelet desialylation primarily encompass aging under normal physiological conditions, autoimmune system disorders in pathological circumstances, exposure to foreign antigens and autoantigens, and assaults by pathogen-derived NEUs. These elements collectively play a significant role in the generation of platelet desialylation (**Figure 2**).

The factors contributing to platelet desialylation are diverse and intricate among which are (i) Aging platelets: Under normal physiological conditions, aging platelets shed sialic acid residue from the surface glycoproteins during circulation. This exposes the subterminal β-Gal, rendering targets for recognition, and clearance by the AMR. This process also induces an up-regulation of TPO, a significant factor in platelet desialylation (33). The influence on platelet desialylation is noteworthy; (ii) Abnormal disorders of the autoimmune system: In pathological states, autoimmune system disorders in ITP patients result in the generation of corresponding antibodies such as anti-GPIIb/IIIa, anti-GPIbα, anti-GPIb/IX and anti-GPIa/IIa antibody. These antibodies may induce platelet desialylation, subsequently cleared by excessive phagocytosis by the monocyte-macrophage system (38–40). The impact of abnormal autoimmune system disorders on platelet desialylation is substantial; (iii) Viral and bacterial infections: Plays a crucial role in platelet desialylation. Antigens carried by viruses, combined with native platelet antigens, lead to cross-activation of T and B cells, epitope spreading, and eventually platelet desialylation (40). Concurrently, NEUs originating from pathogens modify the sialic acids on platelet surfaces, triggering platelet desialylation (37). NEUs, characterized by a mushroom-like tetrameric glycoprotein structure, are prevalent in viruses, bacteria, and mammalian cells (41). NEUs are categorized into four types ranging from NEU1 to NEU4 (**Table 1**). Among them, NEU1, NEU2, and NEU4 are associated with platelet desialylation and are predominantly found on resting platelets. Specifically aggregated by GPIbα activated by vWF, these enzymes catalyze the sialic acid moiety, cleaving the sialic acid residues at the end of the sialic acid glycoprotein complex. This process induces platelet desialylation, creating a positive feedback loop of “platelet activation-desialylation”, ultimately resulting in platelet clearance in the liver (52). Hence, the inhibition of NEUs in the pathogenesis of ITP may contribute to thrombocytopenia. Reducing viral and bacterial infections, along with inhibiting NEUs during the pathogenesis of ITP, holds the potential for ameliorating thrombocytopenia.

**Table 1:** Types of Sialidases.

Categorization	Position	Substrate	Function	References
NEU1	Lysosome	Oligosaccharide, glycopeptides	Phagocytosis, lysosomal degradation, cytotoxicity, immunity	(42–44)
NEU2	Cytoplasm	Oligosaccharide, glycoprotein, gangliosides	Differentiation of myoblasts and neural cells	(42, 45, 46)
NEU3	Cytoplasmic membrane	Gangliosides	Differentiation, apoptosis, and adhesion of neural cells	(47–49)
NEU4	Endoplasmic reticulum, lysosomes, mitochondria	Oligosaccharide, glycoprotein, gangliosides	Differentiation, apoptosis, and adhesion of neural cells	(42, 50, 51)

The aforementioned studies suggest a crucial role for immune factors in platelet desialylation, with abnormalities in the autoimmune system identified as a primary predisposing factor for this phenomenon. Furthermore, specific viral and bacterial infections, along with the aging platelets, have been linked to platelet desialylation. In conclusion, a thorough exploration of these predisposing factors is essential for shedding light on the role of platelet desialylation in the pathogenesis of ITP.

## 4 The process of generation of platelet desialylation

**Figure 3:**
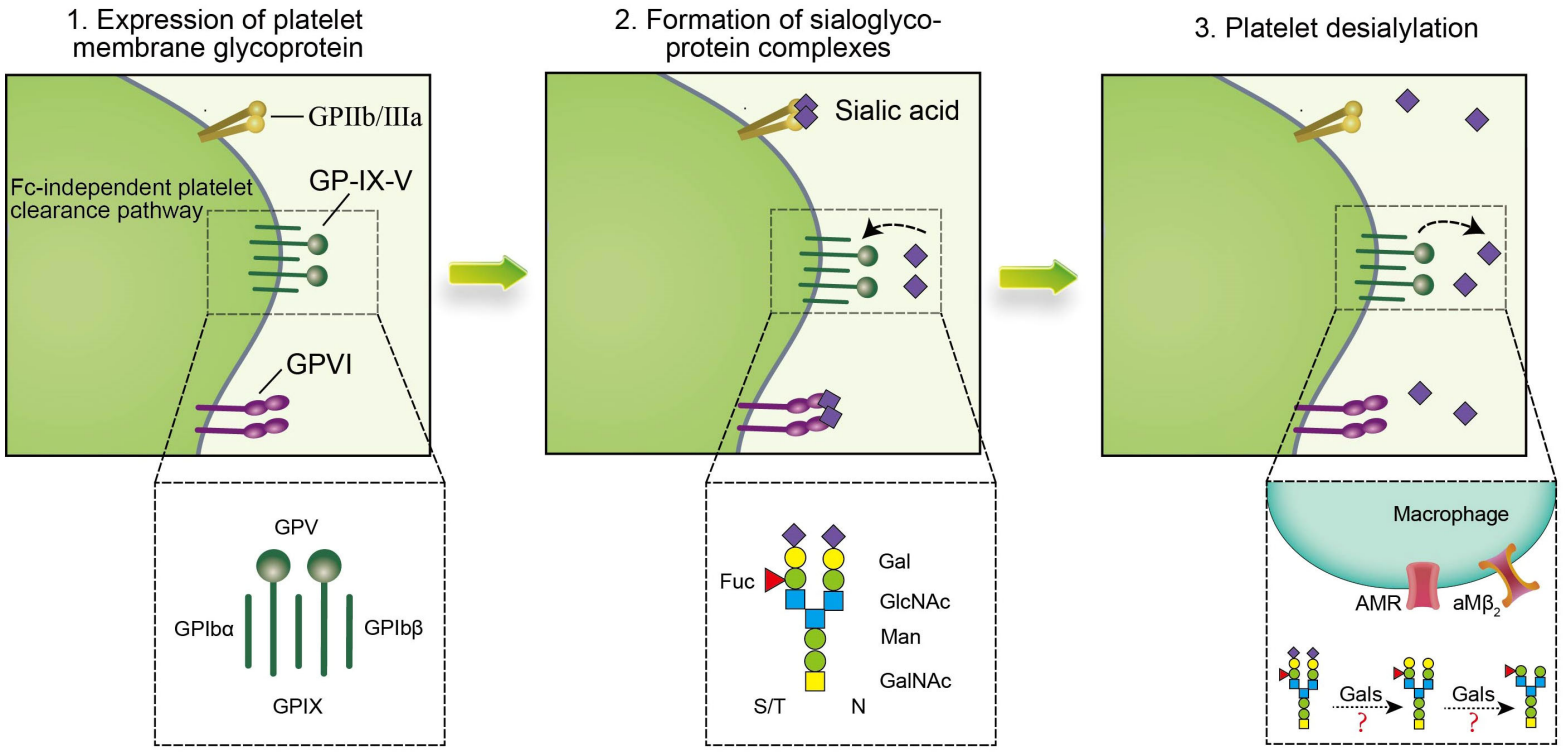
Platelet desialylation involves three key steps: 1. Expression of anti-platelet antibodies, such as anti-GPIIb/IIIa antibodies, anti-GP-IX-V antibodies and anti-GPVI antibodies, in which anti-GPIbα antibodies clear platelets through the Fc-independent platelet clearance pathway, thereby activating immune cells and affecting platelet function and survival. In addition, the abnormal expression or mutation of these anti-platelet antibodies may lead to thrombocytopenia and impaired function, and promote the excessive clearance of platelets by the immune system, thus exacerbating the disease of ITP; 2. Specific binding sites (such as β-Gal and N-GlcNAc) on the terminal sugar chains of platelet surface glycoproteins form sialoglycoprotein complexes with sialic acid molecules to change the structure and function of platelet glycoproteins, thereby affecting platelet biological activity and function; 3. Gals may mediate platelet desialylation, resulting in removal of terminal sialic acid, thereby exposing β-Gal and N-GlcNAc on the surface of platelets, allowing them to be recognized and cleared by specific receptors of the immune system such as AMR and αMβ_2_. Together, these steps constitute the complex mechanism of platelet desialylation in ITP, and its abnormality may lead to impaired and reduced platelet function, which in turn affects the blood coagulation process.

Platelet desialylation is a sophisticated biochemical process comprised of three pivotal stages (**Figure 3**). Initially, there is an expression of relevant antiplatelet antibodies like GPIa/IIa, GPIbα, and GPIb/IX. Subsequently, specific antibodies interact with sialic acid molecules via their specific domains, forming sialic acid-glycoprotein complexes that facilitate mutual recognition and adhesion of platelets to other cells or molecules. Ultimately, platelet desialylation is triggered by NEUs, which removes the terminal sialic acid residues from the sialic acid-glycoprotein complexes. This process leads to the identification of desialylated platelets, subsequently recognized and cleared by the AMR through an Fc-independent platelet clearance pathway.

### 4.1 Expression of antiplatelet antibodies

The expression of antiplatelet antibodies represents an initial step in the process of platelet desialylation, and their levels significantly correlate with the pathogenesis of ITP. These antibodies encompass class I antibodies targeting human leukocyte antigens and those directed at glycoproteins. Notably, glycoproteins targeting antibodies are predominantly localized within the megakaryocyte system, exhibiting specificity as autoantibodies.

Categorized based on the antigenic target site, these antibodies include various types such as anti-GPIa/IIa, anti-GPIbα, anti-GPIb/IX, and anti-GPIIb/IIIa antibodies (53, 54). In the majority of ITP patients, anti-GPIIb/IIIa antibodies are present in 70% to 80% of cases, while anti-GPIbα antibodies are found in 20% to 40% of cases. A minority of ITP patients may have both or exhibit antibodies against other glycoproteins (38). Among these, the anti-GPIIb/IIIa antibody primarily mediates platelet clearance through the FcγR pathway. The binding of GPIIb/IIIa to the Fc segment of its specific antibody activates the macrophage’s tyrosine kinase, binding to the low-affinity FcγRIIA or FcγRIIIA on the macrophage’s surface. This results in platelet removal through both direct phagocytosis and complement-mediated phagocytosis in the spleen (7, 55).

In contrast, most studies reported that anti-GPIbα antibodies predominantly utilize an Fc-independent pathway for platelet clearance. When GPIbα binds to the F(ab)_2_ segment of its specific antibody, it further stimulates platelet activation through the GPIbα-mediated signaling pathway. This induction leads to the removal of sialic acid residues from the platelet surface, exposing subterminal β-Gal, which is recognized and cleared by AMR (24, 56, 57). Overall, anti-platelet antibodies such as GPIbα play a critical role in platelet desialylation. Abnormal expression or mutation of these antibodies can result in thrombocytopenia and impaired function, contributing to the immune system’s excessive clearance of platelets and exacerbating the condition of ITP.

### 4.2 Formation of sialic acid-glycoprotein complexes

The formation of sialic acid-glycoprotein complexes refers to the binding of specific glycoproteins on the surface of platelets to sialic acid molecules. This process significantly influences platelet function and biological activity, and it is a key mechanism in the desialylation of platelets in ITP. During the pathogenesis of ITP, specific binding sites on the terminal glycan chains of platelet glycoproteins, such as β-Gal and N-GlcNAc, can bind to sialic acid molecules, forming “sialic acid-glycoprotein complexes”.

This complex formation alters the structure and function of the platelet glycoproteins, thereby impacting the biological activity and lifespan of platelets within the body. Taking GPIIb/IIIa (αIIbβ_3_) and GPIb-IX-V as examples, these glycoproteins play central roles in both physiological and pathological processes of platelets. GPIIb/IIIa, a key factor in platelet adhesion and aggregation (58, 59), and GPIb-IX-V, which serves as a receptor for vWF and is involved in platelet adhesion and activation following vascular injury, are frequently targeted (60, 61). After binding with sialic acid, these glycoproteins may influence the recognition and clearance mechanisms of platelets, contributing to the pathogenesis of ITP. It has been discovered that GPIIb/IIIa promotes NEU1 surface translocation and platelet desialylation in GPIIb/IIIa-containing ITP patients relies on FcγRIIA signaling on the surface of splenic macrophages rather than the platelet activation pathway (7).

Thus, understanding the binding mechanism between platelet surface glycoproteins and sialic acid, namely, the formation of sialic acid-glycoprotein complexes, is crucial for exploring the role of platelet desialylation in the pathogenesis of ITP.

### 4.3 Platelet desialylation

Initially, galactosidases (Gals) may catalyze the removal of sialic acid residues from the terminal end of the sialic acid-glycoprotein complex, initiating the process of platelet desialylation. Subsequently, desialylation leads to the sequential exposure of β-Gal and N-GlcNAC on the sialic acid-glycoprotein complex, which is then recognized and cleared by the AMR or αMβ_2_ in the liver. Ultimately, deglycosylation results in the exposure of N-GlcNAC, which may be recognized and cleared through carbohydrate receptors, further mediating their uptake by macrophages.

Notably, the AMR, the principal lectin expressed on hepatocytes, is a highly conserved transmembrane hetero-oligomeric glycoprotein complex. The AMR recognizes, and scavenges circulating desialylated platelet glycoproteins (62). It has been established that the AMR facilitates platelet clearance by binding β-Gal, triggering the production of hepatic TPO via the JAK2-STAT3 pathway (33). While the AMR does regulate platelet clearance to a certain degree, the mechanisms by which it participates in the clearance of desialylated platelets have not been fully elucidated. Further research is needed to explore these mechanisms in greater detail.

Additionally, the integrin αMβ_2_ (also known as CD11b/CD18, CR3, or MAC-1), situated on the surface of Kupffer cells, is another receptor that recognizes and binds desialylated platelets as large clusters of non-covalently bound heterodimeric transmembrane glycoproteins. It has been discovered that Kupffer cells can rightfully identify and engage in the phagocytosis of desialylated platelets through αMβ_2_ (63). Furthermore, cold-degalactosylated platelets are cleared by the MAC-1. Recently, it was revealed that macrophage galactose lectin (MGL) from Kupffer cells aids in the clearance of desialylated platelets through collaboration with the AMR (62). These findings imply that platelet desialylation is a crucial element of the “glycan-agglutinin” mechanism, referring to platelet clearance mediated by glycan-agglutinin binding (64, 65).

In summary, the desialylation process of platelets encompasses several crucial stages, among which anti-GPIIb/IIIa antibodies and anti-GPIbα antibodies may be expressed, which play a significant role in the activation and aggregation of platelets. Furthermore, the formation of the sialic acid-glycoprotein complex and platelet desialylation are indispensable in the pathogenesis of ITP. Aberrations in these processes have the potential to lead to desialylation and structural disarray of platelets, thereby impacting the coagulation cascade.

## 5 Molecular regulatory mechanisms of platelet desialylation in ITP

**Figure 4:**
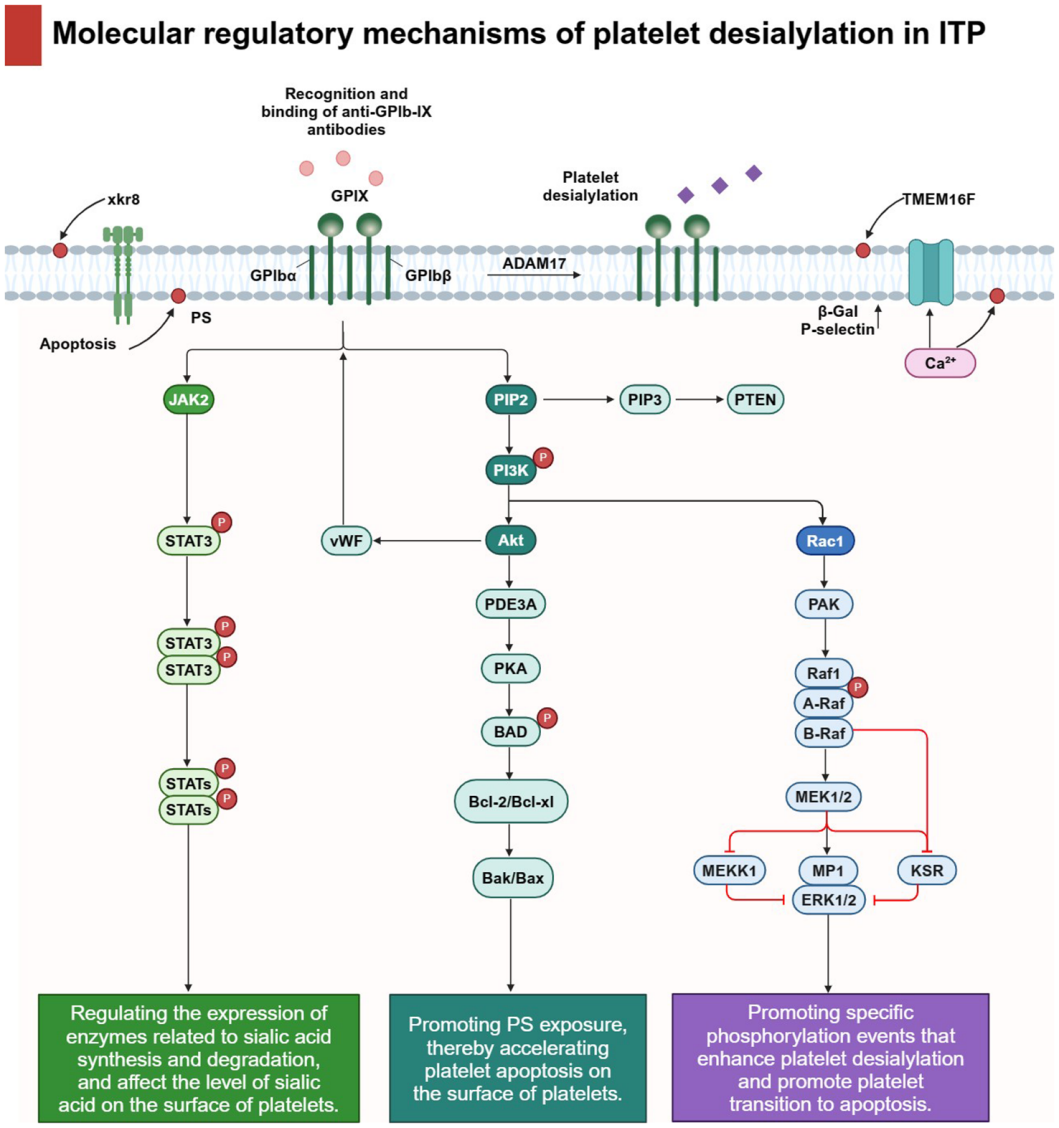
Molecular regulatory mechanisms of platelet desialylation in ITP: The molecular regulatory mechanism of platelet desialylation involves the regulation of several key signaling pathways. In particular, the recognition and binding of anti-GPIb-IX antibodies triggers cascade activation of downstream signals, thereby regulating multiple signaling pathways closely related to platelet function. For example, the PI3K/AKT signaling pathway plays a central role in platelet desialylation, accelerating platelet apoptosis by inhibiting PKA activity and promoting PS exposure. Secondly, JAK/STAT signaling pathway directly affects the level of sialic acid on the surface of platelets and accelerates the degradation process of sialic acid by regulating the expression of enzymes related to sialic acid synthesis and degradation. Finally, activated MAPK signaling pathways enhance platelet desialylation and promote platelet transition to apoptosis by promoting specific phosphorylation events. Activation of these signaling pathways not only promotes biochemical changes in desialylation, but also leads to significant changes in platelet function. Under the action of metalloproteinase ADAM17, the sialic acid on the cell surface is clipped and activates the anti-apoptotic signaling pathway within the cell by interacting with receptors on the cell surface. In addition, through the synergic action of calcium-activated TMEM16F and xkr8 molecules, PS is redistributed from the inner lobules of the plasma membrane to the outer lobules, and this change further leads to increased expression of β-Gal and P-selectin, which intensifies platelet activation and clearance.

The molecular regulatory process of platelet desialylation involves the coordinated regulation of several key signaling pathways, including PI3K/AKT, JAK/STAT, and MAPK signaling pathways (**Figure 4**). A comprehensive understanding of these signaling pathways and their interconnections is crucial to grasp the role of platelet desialylation in the pathogenesis of ITP.

### 5.1 PI3K/AKT signaling pathway

The PI3K/AKT signaling pathway is a critical cellular pathway with significant implications for the pathogenesis of ITP, playing a pivotal role in the generation of platelet desialylation.

In the context of platelet desialylation, the PI3K/AKT signaling pathway contributes to maintaining platelet stability, inhibiting apoptosis, and promoting cell survival. Activation of PI3K kinase leads to the generation of the intracellular secondary signaling molecule PIP3, subsequently activating the AKT kinase. Activated AKT then mediates platelet desialylation through various pathways. Research has suggested that the decreased platelet counts observed in ITP patients with anti-GPIbα antibodies may be attributed to the activation of the PI3K pathway induced by the anti-GPIbα antibody, leading to AKT activation, a downstream effector of PI3K (66). Subsequently, AKT induces platelet apoptosis by diminishing the activity of protein kinase A (PKA), regulated by phosphodiesterase (PDE3A), and simultaneously instigates platelet activation through the AKT pathway. The exposure of apoptotic and activated platelets to phosphatidylserine (PS) on the membrane surface allows recognition, phagocytosis, and subsequent elimination of platelets by Kupffer cells, resulting in decreased platelet count. Suppression or genetic ablation of AKT or AKT-regulated apoptotic signaling, or the obstruction of PS exposure, can protect platelets from clearance. Inhibiting the biological activity of PDE3A, PKA, PS, etc., or conducting gene knockdown of relevant proteins could potentially mitigate antibody-induced platelet removal and augment the platelet count. Another investigation revealed that Akt not only interacts with the cytoplasmic structural domain of GPIbα but also transmits vWF-GPIbα interaction signaling, culminating in platelet activation (67, 68). Furthermore, the PI3K/AKT signaling pathway may potentially interact with other crucial signaling pathways to accelerate platelet desialylation. For instance, the interaction of AKT with other proteins in the PI3K signaling pathway could trigger the release of calcium ions from platelets and activate the Transmembrane Protein 16F (TMEM16F), thereby influencing the aggregation and adhesion properties of platelets (66, 69). Consequently, the suppression of AKT-mediated apoptosis mitigates platelet clearance *in vivo*, presenting a potential novel therapeutic approach for treating ITP.

These findings underscore the pivotal regulatory function of the PI3K/AKT signaling pathway in platelet desialylation, thereby impacting the pathogenesis of ITP through its influence on platelet activation and apoptosis.

### 5.2 JAK/STAT signaling pathway

In the pathogenesis of ITP, the JAK/STAT signaling pathway may regulate platelet desialylation through diverse mechanisms. Initially, its activation may potentially influence the expression of key enzymes involved in sialic acid synthesis, consequently leading to reduced sialic acid production. Subsequently, aberrant JAK/STAT signaling may enhance sialidase activity, accelerating sialic acid degradation and resulting in reduced sialic acid on the platelet surface. Ultimately, this pathway may also govern the expression of genes associated with the biosynthesis and degradation of sialic acid, thereby adjusting the concentration of sialic acid on the platelet surface at the transcriptional level, consequently influencing the process of platelet desialylation. Observations indicate that the AMR interacts with desialylated platelets, modulating the production of TPO via the JAK2 signaling pathway and STAT3 activation, both *in vitro* and *in vivo* (33, 70). This discovery offers new perspectives and a deeper understanding of the prevalent adverse clinical effects associated with JAK1/2 inhibition in ITP. Another study found that IL-1β could induce an autocrine loop of IFN-β in hematopoietic cells, activating the JAK/STAT signaling pathway and further mediating the surface translocation of NEU1 on MKs in patients with ITP, leading to platelet desialylation and decreased platelet production. The JAK1/2 inhibitor Baricitinib has demonstrated its ability to reverse platelet desialylation and dysfunction both *in vitro* and *in vivo* (71).

The above-mentioned results highlight the significant function of the JAK/STAT signaling pathway in platelet desialylation. It not only regulates the signaling aspects of platelet desialylation but also influences the sialic acid content on the platelet surface by modulating NEU expression.

### 5.3 MAPK signaling pathway

In platelets, the MAPK signaling pathway serves as a regulator for various cellular processes, including platelet differentiation and apoptosis. Consequently, this pathway might also be associated with platelet sialic acid metabolism and desialylation. Chen et al. observed significantly elevated expression levels of MAPK and mTOR, along with their phosphorylated proteins, in the platelets of ITP patients compared to healthy individuals. This suggests that the activation of the MAPK signaling pathway in the platelets of ITP patients might be associated with platelet desialylation and apoptosis (72). Furthermore, the activation of cytoplasmic phospholipase A2, mediated by p38 MAPK, resulted in the release of arachidonic acid from membrane phospholipids, causing the shedding of 14–3-3ζ from Bad. Subsequently, 14–3-3ζ, bound to the cytoplasmic region of GPIbα, initiates platelet desialylation and apoptosis (73, 74). The aforementioned findings indicate that the MAPK signaling pathway could play a pivotal role in the process of platelet desialylation, providing valuable insights into the modulating of this process and enhancing our understanding of the pathogenesis of ITP. However, the precise influence of this signaling pathway on the process of platelet desialylation remains to be clarified, necessitating further research in the future.

In conclusion, the molecular regulatory mechanisms of platelet desialylation involve the modulation of multiple signaling pathways. These regulatory mechanisms are crucial for maintaining platelet stability, functionality, and lifespan. Aberrant activation or impairment of these signaling pathways could lead to a decrease in platelet count and abnormal function. Hence, a comprehensive exploration of the molecular regulatory mechanisms of platelet desialylation is anticipated to provide new pathways and strategies for the accurate diagnosis and personalized therapy of ITP.

## 6 The potential application of sialylation modulation strategies in the treatment of ITP

The treatment strategy for ITP should adhere to the principles of stratification and personalization to ensure sustained platelet counts at safe levels and reduce incidents of bleeding while minimizing adverse effects. Glucocorticoids remain the primary therapeutic agent for ITP. However, a staggering 98% of patients undergoing prolonged hormonal therapy experience side effects, leading to 38% discontinuing or reducing their medication due to intolerance. Additionally, some patients exhibit no response to glucocorticoids or experience a disease relapse upon cessation of hormonal treatment (75, 76). Furthermore, certain strategies such as the administration of rituximab (77, 78) and splenectomy (79, 80) aim to manage ITP by reducing platelet destruction. However, there remains a subset of patients for whom these treatments are ineffective or who encounter postoperative recurrence issues (81). In recent years, the emergence of sialidase inhibitors has opened up novel therapeutic avenues for ITP patients, showing enhanced efficacy in clinical management (**Table 2**). Currently, primary sialidase inhibitors include Oseltamivir and 2-deoxy-2,3-didehydro-N-acetylneuraminic acid (DANA), which may enhance platelet production by inhibiting platelet desialylation, consequently augmenting platelet counts in ITP patients.

**Table 2:** Therapeutic Effect of Sialidase Inhibitors.

Medicines	Object	Research design	Method of administration	Results of treatment	*P-value*	References
Oseltamivir	A 13-year-old girl with chronic ITP	–	Oral Oseltamivir 75 mg twice daily for 5 days with 30 days follow-up	After 16 days of Oseltamivir treatment, the patient’s platelet counts increased from 4 × 10^9^/L to 97 × 10^9^/L	–	(82)
Oseltamivir	A cohort of 10 female patients aged 5–79 years with chronic ITP	–	Oral Oseltamivir 75 mg twice daily for 5 days with a 6-month follow-up	After 6 months of Oseltamivir treatment, the patient exhibited a 40% response rate, and platelet counts increased from 16×10^9^/L to 111×10^9^/L	–	(83)
Oseltamivir	385 adult patients with influenza A or B	Retrospective study	Oral Oseltamivir 0.99 mg (cumulative dose)	After 14 days of Oseltamivir administration, the two groups of patients receiving Oseltamivir demonstrated increased platelet counts of (57.53 ± 93.81) ×10^9^/L, *p*=0.013, and (50.79 ± 70.59) ×10^9^/L, *p*=0.023, respectively. These counts were significantly higher than those in the group without Oseltamivir, which remained at (18.45 ± 89.33) ×10^9^/L	*P*<0.05	(84)
Oseltamivir	35 individuals diagnosed with ITP spanning ages 2 to 79 years	Multicenter, prospective study	Oral Oseltamivir 75 mg twice daily for 5 days with a 3-month follow-up	After 3 months of Oseltamivir treatment, the overall response rate was 66.7% (3 CR, 1 R)	*P*<0.05	(85)
Dexamethasone combined with Oseltamivir	A group of 96 patients aged 18 years or older with newly diagnosed untreated ITP	Multicenter, randomized, open-label, parallel groups	Dexamethasone monotherapy (40 mg/day orally for 4 days) or combination therapy with dexamethasone (40 mg/day orally for 4 days) plus Oseltamivir (75 mg orally twice daily for 10 days) was administered, followed by a 6-month follow-up period.	After 14 days of administering Oseltamivir, patients in the Oseltamivir-co-dexamethasone group showed a notably higher initial remission rate (86%) compared to the decemethasone-only group (66%; odds ratio (OR) 3.18; 95 CI% 1.13–9.23). Furthermore, after 6 months of treatment, the rate of sustained remission was significantly higher in the Oseltamivir combined with dexamethasone group than in the dexamethasone-only group (23 (53%) vs. 14 (30%); OR 2.17; 95% CI 1.16–6.13)	*P*<0.05	(86)
Oseltamivir	A set of 7 female patients aged 16–74 years with chronic, persistent, or refractory ITP	Prospective, single-group intervention study	Oral Oseltamivir 75 mg twice daily for 5 days with a 6-month follow-up	After 6 months of Oseltamivir treatment, 3 patients (42.9%) remained in remission and 1 patient developed a Complete Response (14.3%)	*P*<0.05	(87)

Oseltamivir is a specific sialidase inhibitor widely utilized in clinical settings for the prophylaxis and therapy of Influenza A or B virus infection, stands out among these inhibitors (88). Compared to existing ITP treatment drugs, Oseltamivir mitigates the immune cells’ onslaught on platelets by interfering with the structure of sialic acids on their surface. Meanwhile, this oral therapy is clinically readily available and more cost-effective compared to intravenous immunoglobulin therapy and most second-line therapies. Furthermore, re-administration of Oseltamivir during relapse resulted in higher remission rates. Multiple independent retrospective studies indicate that Oseltamivir may mitigate the destruction of peripheral platelets by inhibiting platelet desialylation and restoring platelet counts in ITP patients (82–84, 89, 90). Combining Oseltamivir with other therapeutic approaches has the potential to optimize current first-line therapy, potentially enhancing platelet responses to improve the long-term prognosis of ITP patients. In a clinical study, four out of six patients with refractory ITP who were administered Oseltamivir in conjunction with other ITP medications achieved remission and exhibited higher initial response rates (85). A recently published Phase II trial investigating the efficacy of Oseltamivir coupled with dexamethasone for treating ITP in *Lancet Hematology* indicated that the group treated with the Oseltamivir-dexamethasone combination demonstrated significantly higher effectiveness in treating ITP compared to the group treated with dexamethasone alone (86). Although additional studies have explored the potential of Oseltamivir as a novel target therapy in ITP, more comprehensive large-scale clinical trials are required to systematically assess its long-term effectiveness and side effects (such as nausea, vomiting, headache, muscle pain and drowsiness). Substantial evidence-based medicine is needed to validate the safety and effectiveness of Oseltamivir as a first-line therapeutic choice for ITP.

DANA, a synthetic inhibitor of mammalian, bacterial, and viral sialidases, has been investigated as a potential therapeutic agent for ITP. Several *in vivo* experiments utilizing a mouse model of ITP showed that DANA effectively decreased platelet breakdown by inhibiting platelet desialylation (91, 92). These promising results suggest that DANA holds potential as a viable therapeutic strategy for ITP, providing valuable insights for future clinical investigations and the advancement of related pharmaceuticals.

The aforementioned findings propose that aberrant desialylation alterations in ITP could present novel therapeutic targets. Consequently, inhibiting desialylation using sialidase inhibitors is expected to emerge as a fresh therapeutic approach for treating patients diagnosed with ITP. However, further clinical investigations are necessary to confirm the safety and long-term efficacy of these medications, as well as to determine the optimal methods for their utilization.

## 7 Conclusions and perspective

The classical mechanisms of ITP mainly involve autoantibodies and the destruction of platelets mediated by the immune system (93, 94). However, recent research indicates that the Fc-independent pathway of platelet clearance also plays a part in the pathogenesis of ITP, with desialylation recognized as a new mechanism for Fc-independent platelet clearance. NEUs facilitate platelet desialylation, leading to Fc-independent platelet clearance in patients with ITP via the AMR.

Given the complexity of the biological processes driving the pathogenesis of ITP, potential directions for future research towards mitigating platelet desialylation could include the following four areas: (1) reducing the aging of circulating platelets and the stimulation of the immune system due to viral and bacterial infections and their associated antigens; (2) regulating the expression of antiplatelet antibodies and the formation of the sialic acid-glycoprotein complex to prevent platelet reduction and dysfunction caused by their abnormal expression or mutation; (3) through comprehensive research of platelet desialylation, exploring specific targets and conducting subsequent experiments to regulate the degree of platelet desialylation using AMR inhibitors and gene knockout techniques, to reduce the production of antiplatelet antibodies; (4) identifying specific biomarkers associated with platelet desialylation, such as those that could modulate NEU activity based on variations in platelet counts.

In conclusion, the process of platelet desialylation significantly impacts the pathogenesis of ITP. Therefore, a comprehensive exploration of the factors causing platelet desialylation, the underlying biochemistry and molecular regulation of this process, and the application of desialylation regulation strategies for the treatment of ITP may help develop new therapeutic approaches and techniques for this disease. It will also facilitate the accurate diagnosis and individualized treatment of ITP patients to enhance treatment effectiveness and improve their overall well-being while reducing the burden of immune-related platelet disorders on the healthcare system.

## Author contributions

QZ: Conceptualization, Investigation, Methodology, Writing – original draft. MH: Conceptualization, Investigation, Validation, Writing – original draft. ET: Validation, Writing – review & editing. LW: Investigation, Validation, Writing – review & editing. JAL: Investigation, Validation, Writing – review & editing. XL: Methodology, Software, Writing – review & editing. JSL: Software, Supervision, Writing – review & editing. WZ: Conceptualization, Supervision, Writing – review & editing. JW: Conceptualization, Funding acquisition, Supervision, Writing – review & editing.
